# A team approach to providing refractive error services

**Published:** 2014

**Authors:** Kovin Naidoo, Pirindha Govender

**Affiliations:** Global Programmes Director: Brien Holden Vision Institute, Durban, South Africa.; Global Programmes Associate: Brien Holden Vision Institute, Durban, South Africa.

**Figure:**
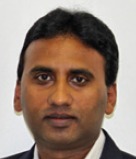
Kovin Naidoo

**Figure:**
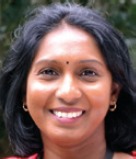
Pirindha Govender

Worldwide, there are over 640 million people who are vision impaired, simply because they do not have access to a simple eye examination and a pair of spectacles.^1^ With 43% of vision impairment being due to uncorrected refractive error,^1^ it is no wonder that there have been increased efforts to improve service delivery in this area. However, a recipe for successfully and predictably ‘scaling up’ (expanding) programmes to provide eye examinations and spectacles to everyone in need has thus far remained elusive.

There have been different configurations used when expanding refractive error services, some of which have seen optometrists and refractionists integrated as core members of the eye care team, and others in which they have worked outside this team. Regardless of the configuration, we believe that a team approach to refractive error care will create a collaborative and enabling environment which will ultimately benefit patients.

In a team approach (Figure 1), personnel at the community level – such as community health workers – can conduct health promotion and screening activities to encourage individuals to seek eye examinations for refractive error. It will also detect those who need to be referred. At the primary level (eye clinic), personnel such as nurses can screen and separate refractive from non-refractive patients (the pinhole is particularly useful in this respect), and provide presbyopic correction for those whose vision impairment is not caused by distance refractive error or ocular disease. At the secondary level, comprehensive refractive examinations should be provided by optometrists, ophthalmic clinical officers and other mid-level personnel trained for this purpose. Ophthalmologists should be deployed at this level in cases where they are the primary refractive personnel in the country. At the tertiary level, pre- and post-operative refraction of patients, management of conditions such as keratoconus, and other medical-related contact lens fitting can be provided by optometrists in a co-management agreement with ophthalmologists. These personnel will also work closely with specialised clinics such as advanced low vision services or rehabilitation services.

**Figure:**
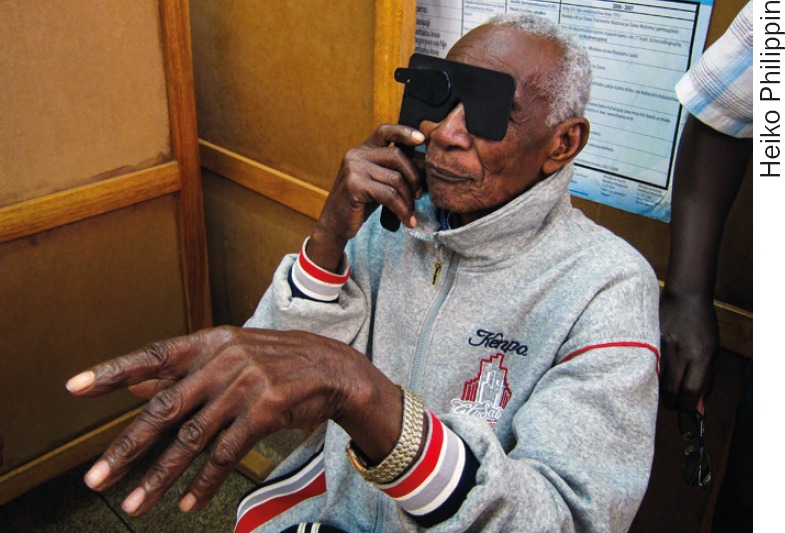
Pinhole testing can be carried out by nurses or other primary health care workers to detect patients who need refractive error care

A team approach to refractive error care ensures that eye health workers all identify themselves as part of a team. Each team member's role must be clearly defined by the needs of the health system within which they work, while maintaining their primary professional role. Consideration must be given to the complementary nature of the job of each member and their inter-dependency in the team.^1^

Adopting a team approach to eye care helps to optimise staff experience, knowledge and skills. For example, as optometry is increasingly being integrated at regional and district hospitals, there is an opportunity to shift tasks like refraction, low vision, ocular disease screening, pre-operative assessment and post-operative follow-up examinations to optometrists (or to ophthalmic technicians or ophthalmic nurses, where they are available). This will free ophthalmologists to focus on surgery and the management of disease.

Some aspects of refractive services may be ‘task shifted’ to others, e.g. nurses screening for myopia, hyperopia and presbyopia. Prescribing presbyopia spectacles for individuals who have good distance vision and no obvious pathology could take place at this level, and appropriate referral protocols should be defined.

A team approach and task shifting requires the eye care system to provide the appropriate training required by different health workers so that a good quality service can be provided at all levels, and more patients can be seen.

One example of a flexible training approach is provided by the Regional School of Optometry in Malawi, set up by a partnership comprising the Brien Holden Vision Institute, SightSavers and Optometry Giving Sight. It consists of two programmes:

- a conventional four-year BSc optometry degree that trains individuals for public and private sector deployment.
- an optometric training diploma which is delivered over 3 years and allows graduates to provide refractive services and eye care in the public sector, where the need is greatest.

Graduates of the diploma programme have the opportunity to upskill and progress to the four-year degree, thereby meeting both the professional needs of the person and the needs of society.

**Figure:**
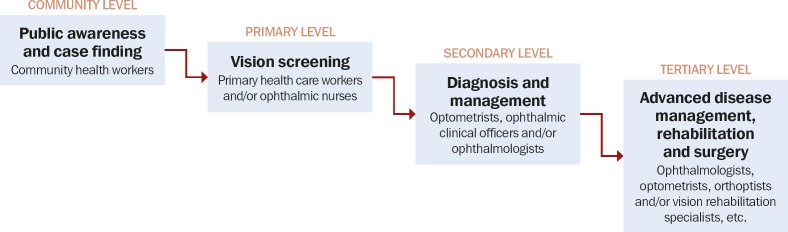
Figure 1. One example of how members of a refractive error team could work together

Although each setting is different, a team approach rooted in the local human resources for eye health strategy and health policies will help to make services more sustainable. Ideally, decisions should be informed by evidence-based, context-specific research to determine feasibility and impact. One example is the ‘Giving Sight to KZN’ project supported by Standard Chartered Bank. This involved the integration of refractive error services into the district health system in KwaZulu-Natal, South Africa.^2^

Whatever the situation, we should not compromise on a team approach and should instead actively seek integration with other components of the health system. The very success of our efforts depends on this.

**Professional competition**

The public health challenges in low-and middle-income countries, where the eye care needs are greatest, demand of us a collaborative and partnership approach.

Professional competition within refractive error service delivery may be positive. Appropriately trained cadres who are supported with continuing professional development are able to increase the professional standard of the services they provide. Overtime, better and more professional services increase community expectation, driving a need for increasing professional competency among practitioners in a given geographic area. We cannot, however, allow the professional battles that sometimes occur in high-income countries to intrude upon our work in low- and middle-income countries, or allow the narrow interests of professionals to dominate the service delivery landscape.

The roles of different eye service providers (ophthalmologists, optometrists, ophthalmic nurses, ophthalmic clinical officers, and other allied personnel) should be defined by a collective evaluation of the needs of a particular eye health system, the distribution and availability of the different types of provider and the potential to task shift at all levels.

There are enough poor people to reach in our world; it is unnecessary for us to trip over each other to serve them.
